# Partial catalytic Cys oxidation of human GAPDH to Cys-sulfonic acid.

**DOI:** 10.12688/wellcomeopenres.15893.2

**Published:** 2020-08-25

**Authors:** Andrea Lia, Adam Dowle, Chris Taylor, Angelo Santino, Pietro Roversi

**Affiliations:** 1 Leicester Institute of Chemical and Structural Biology and Department of Molecular and Cell Biology, University of Leicester, Henry Wellcome Building, Lancaster Road, LE1 7HB, UK; 2 Institute of Sciences of Food Production, C.N.R. Unit of Lecce, ia Monteroni, Lecce, 73100, Italy; 3 Bioscience Technology Facility Department of Biology, University of York, Wentworth Way, York, YO10 5DD, UK

**Keywords:** GAPDH, cysteine S-sulfonic acid, X-ray crystallography, mass-spectrometry, moonlight enzyme

## Abstract

**Background**: n-Glyceraldehyde-3-phosphate dehydrogenase (GAPDH) catalyses the NAD ^+^-dependent oxidative phosphorylation of n-glyceraldehyde-3-phosphate to 1,3-diphospho-n-glycerate and its reverse reaction in glycolysis and gluconeogenesis.

**Methods**: Four distinct crystal structures of human n-Glyceraldehyde-3-phosphate dehydrogenase ( *Hs*GAPDH) have been determined from protein purified from the supernatant of HEK293F human epithelial kidney cells.

**Results**: X-ray crystallography and mass-spectrometry indicate that the catalytic cysteine of the protein ( *Hs*GAPDH Cys152) is partially oxidised to cysteine S-sulfonic acid. The average occupancy for the Cys152-S-sulfonic acid modification over the 20 crystallographically independent copies of *Hs*GAPDH across three of the crystal forms obtained is 0.31±0.17.

**Conclusions**: The modification induces no significant structural changes on the tetrameric enzyme, and only makes aspecific contacts to surface residues in the active site, in keeping with the hypothesis that the oxidising conditions of the secreted mammalian cell expression system result in *Hs*GAPDH catalytic cysteine S-sulfonic acid modification and irreversible inactivation of the enzyme.

## Introduction

Mammalian HEK293F cells are routinely used in conjunction with secreted protein expression vectors for recombinant protein production ^1^. They owe their popularity to ease of handling, robust growth rate, excellent transfectability, high capacity for recombinant protein expression, low-cost media requirements and low levels of secreted contaminants ^2^. Of course, in absence of secretion of the desired recombinant protein at hand, contaminants are the only proteins present in recombinant protein expression systems ^3, 4^. In the course of a research effort aimed at the purification of recombinant *Chaetomium thermophilum* ERAD mannosidase *Ct*HTM1P from the supernatant of HEK293F cells, we purified, crystallised and determined crystal structures of human n-Glyceraldehyde-3-phosphate dehydrogenase ( *Hs*GAPDH , EC 1.2.1.12) ^5^.

GAPDH is essential for glycolysis and gluconeogenesis; it catalyses the NAD+-dependent oxidative phosphorylation of n-glyceraldehyde-3-phosphate to 1,3- diphospho-n-glycerate (and its reverse reaction) ^6^. The GAPDH-catalysed forward reaction occurs at an important transition point in glycolysis between the enzymatic steps that consume and generate ATP. Several studies indicate that the enzyme has pleiotropic functions independent of its canonical role in glycolysis ^7– 9^. In addition to the somatic cells *Hs*GAPDH isoform, the human genome encodes a testis-specific isoform *Hs*GAPDHS (G3PT_HUMAN (Uniprot O14556)), which is expressed only in the post-meiotic period of spermatogenesis. In multiple mammalian species the sperm isozyme (GAPDHS) shares about 70% amino acid identity with the somatic isozyme, and possesses an additional 72-residues N-terminal extension. The latter enables association of GAPDHS to the sperm flagellum fibrous sheath, so that the enzyme – together with other glycolytic enzymes - provides a localised source of ATP that is essential for sperm motility ^10^.

**Figure 1.**
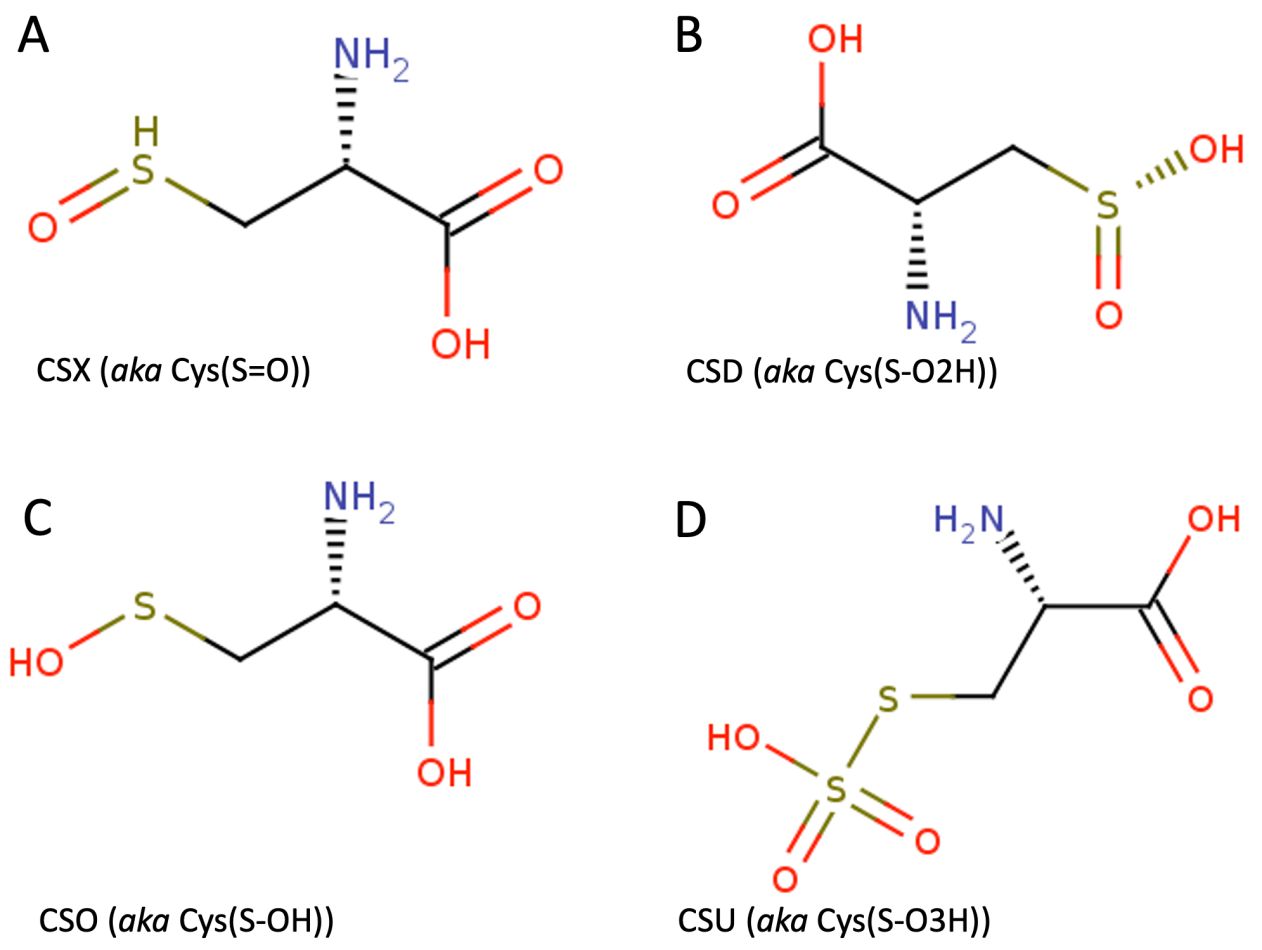
Cys oxidised modifications and GAPDH PDB entries that carry them at the catalytic Cys152. **A**: S-Oxy-Cysteine *aka* Cysteine sulfenic acid (CSX, PDB ID 1J0X); **B**: S-Cysteinesulfinic acid *aka* S-sulfinocysteine *aka* 3-Sulfino-L-alanine (CSD, PDB IDs 2VYN and 2VYV); **C**: S-hydroxy-cysteine (CSO, not described in any GAPDH structures); **D**: Cysteine-S-sulfonic acid (CSU, PDB ID 5M6D and this work)

GAPDH was one of the first enzymes to be crystallised ^11, 12^ and one of the first enzymes whose structure was determined by X-ray crystallography ^13, 14^. A number of studies have reported the GAPDH catalytic Cys carrying oxidised post-translational modifications. In particular, cysteine sulfenic acid (CSX, see Figure 1A) has been observed in the structure of rabbit muscle GAPDH ^15^ (of course, by X-ray diffraction alone, this modification may be difficult to distinguish from S-hydroxy-cysteine (CSO, see Figure 1C)). Cysteine-S-sulfinic acid (CSD, see Figure 1B) has been observed in structures of rat GAPDHS and *E. coli* GAPDH ^16^. Cysteine-S-sulfonic acid (CSU, see Figure 1D) modifies the catalyic Cys in a structure of *Streptococcus pneumoniae* GAPDH ^17^.

In this paper, we present and discuss mass spectrometry and crystallographic evidence supporting partial oxidation of the catalytic Cys residue of *Hs*GAPDH purified from the supernatant of a HEK293F cell culture. A fraction of the molecules shows Cys-S-sulfonic acid instead of Cys in the active site. The four *Hs*GAPDH crystal structures we describe (which we label as P2 _1_ forms A-D, see Table 1) represent novel *Hs*GAPDH monoclinic polymorphs and are the first *Hs*GAPDH crystal structures carrying a Cysteine-S-sulfonic acid modification of the catalytic cysteine Cys152. Crystal form A is the highest resolution *Hs*GAPDH structure determined to date (1.52 Å).

**Table 1.** Human GAPDH crystal structures.

Isoform (Uniprot)	PDB ID	Polymorph (Z)	Resolution (Å)	Tetramer PG	Reference
G3P_HUMAN (P04406)	6YND	P2 _1_ Form A (16)	1.54	1	This work
	6YNE	P2 _1_ Form B (8)	1.85	1	This work
	6YNF	P2 _1_ Form C (16)	2.39	1	This work
	6YNH	P2 _1_ Form D (8)	2.62	1	This work
	1U8F, 1ZNQ	P2 _1_2 _1_2 _1_ Form A (16)	1.75, 2.50	1	^19, 20^
	3GPD	C2 Form A (8)	3.50	2	^21^
	4WNC	P2 _1_ Form E (16)	1.99	1	^22, 23^
	4WNI	P2 _1_2 _1_2 _1_ Form B (16)	2.30	1	^22, 23^
	6IQ6	P2 _1_ Form F (16)	2.29	1	^24^
	6ADE	I222 (24)	3.15	2 and 222	N/A
G3PT_HUMAN (O14556)	3H9E, 3PFW, 5C7O	C2 Form B (8)	1.72, 2.15, 1.73	2	^10, 25^
	5C7O	P3 _1_21 (12)	1.86	2	^10^

## Methods

### Cloning

The cloning of the *Chaetomium thermophilum* ERAD mannosidase *Ct*HTM1P (the product of the gene CTHT_0058730, Uniprot entry G0SCX7_CHATD) in the *Ct*HTM1P _50−1092_-pHLsec vector for secreted mammalian cell expression is described in detail in the Open Laboratory Notebook page (see *Extended data* ^18^). Briefly, the DNA encoding *Ct*HTM1P _50−1092_ was PCR amplified from a commercially obtained plasmid encoding the full length gene, and inserted into the pHLsec vector ^2^ using ligation independent cloning: the *Ct*HTM1P _50−1092_ DNA insert and the AgeI/KpnI linearised ^*AgeI /KpnI*^ pHLsec DNA were mixed in 3:1 molar ratio: 0.06pmol of *Ct*HTM1P _50−1092_ DNA (122 ng) and 0.02pmol of AgeI/KpnI linearised pHLsec DNA (60 ng). To this DNA, 10 *µ*L of Gibson Assembly MasterMix(2X) (New England Bioscience E2611L) were added and the total volume made 20 *µ*L with deionised water. The mix was heated at 50 °C for 1 hour.

### Protein expression

A volume of 450 mL of HEK293F cells at a concentration of 10 ^6^ cells/mL suspended in GIBCO FreeStyle 293 Media (ThermoFisher Scientific 12338018) was transfected with the *Ct*HTM1P _50−1091_-pHLsec vector, using the FreeStyle MAX 293 expression system (Thermo Fisher K900010). Briefly, 1 *μ*g of DNA was used per mL of culture: the DNA vector was initially dissolved in 45 mL of phosphate-buffered saline (PBS: 0.01 M phosphate buffer pH 7.4, 0.0027 M potassium chloride and 0.137 M sodium chloride; PBS tablets, Sigma Aldrich P4417) and vortexed vigorously for 3 seconds; 1.8 mL of a filter-sterilised solution of 0.5 mg/ml polyethylenimine (PEI) was added to the PBS/DNA solution and vortexed vigorously for 3 seconds; the mixture was incubated at room temperature for 20 minutes; the DNA/PEI mixture was added to the cell culture; the cell culture was made 5 *μ*M kifunensine (an inhibitor of endoplasmic reticulum and Golgi mannosidases, Cayman Chemical 109944-15-2). The cell culture was split into three 500 mL Erlenmeyer flasks with 0.2 *μ*m vent caps (Corning), with 150 mL of culture in each flask, and incubated in an orbital shaker incubator at 37°C, shaking at 120 rpm, under a 5% CO _2_ atmosphere. The cells’ supernatant was harvested 4 days post-transfection by centrifuging at 4,000 x g for 5 minutes.

### Protein purification

The cell supernatant was made 1x PBS by addition of the appropriate volume of 10x PBS stock (obtained by dissolving five PBS tablets (Sigma Aldrich P4417) in 200 mL of deionised water). The pH was adjusted to 7.4 and the solution filter-sterilised through a 0.2 *μ*m bottle-top filter. Nickel immobilised metal affinity chromatography (IMAC) was used as the first step of purification. A 5mL HisTrap HP column (GE Healthcare 17-5248-01) was equilibrated with five column volumes (CV) of binding buffer (50 mM sodium phosphate pH 7.5, 300 mM NaCl, 1 mM TCEP (Sigma Aldrich C4706)). The cell culture supernatant was loaded onto the column at room temperature at a flow rate of 3 mL/min. After a 5CV wash in binding buffer, the column was fitted to an ÄKTA purifier fast protein liquid chromatography (FPLC) machine in a 4°C cabinet and further washed with 6 mL of elution buffer (50 mM sodium phosphate pH 7.5, 500 mM imidazole (Honeywell Fluka 56750), 200 mM NaCl).

The wash fractions from the IMAC run (total volume 4.5 mL) were pooled and concentrated to 2 mL in a polyethersulfone spin concentrator of 10 kDa MW cut-off (Thermo Fisher Scientific 88513). The concentrated sample was filtered through a 0.22 *μ*m spin filter and loaded on a 2 mL loop connected to an ÄKTA purifier FPLC machine in a 4°C cabinet. The sample was injected on a size exclusion chromatography (SEC) S200 16/60 column (GE Healthcare 28-9893-35) equilibrated in filter-sterilised and degassed 20 mM HEPES pH 7.5, 100 mM NaCl and 1mM TCEP (Sigma Aldrich C4706) buffer, and run down the column at 0.4mL/min flow rate, collecting 1 mL elution fractions. *Hs*GAPDH-containing fractions were pooled and concentrated as described before to a volume of 70 *µ*L, and protein concentration measured by loading 1.5 *µ*L of sample on a NanoDrop 1000 spectrophotometer (Thermo Scientific). The absorbance was OD _280_=15.0, equivalent to a concentration of 18.8 mg/mL (calculated from ^*HsGAPDH*^ *ϵ*_280_=0.7963 (mg/mL) ^−1^ cm ^−1^).

**Figure 2.**
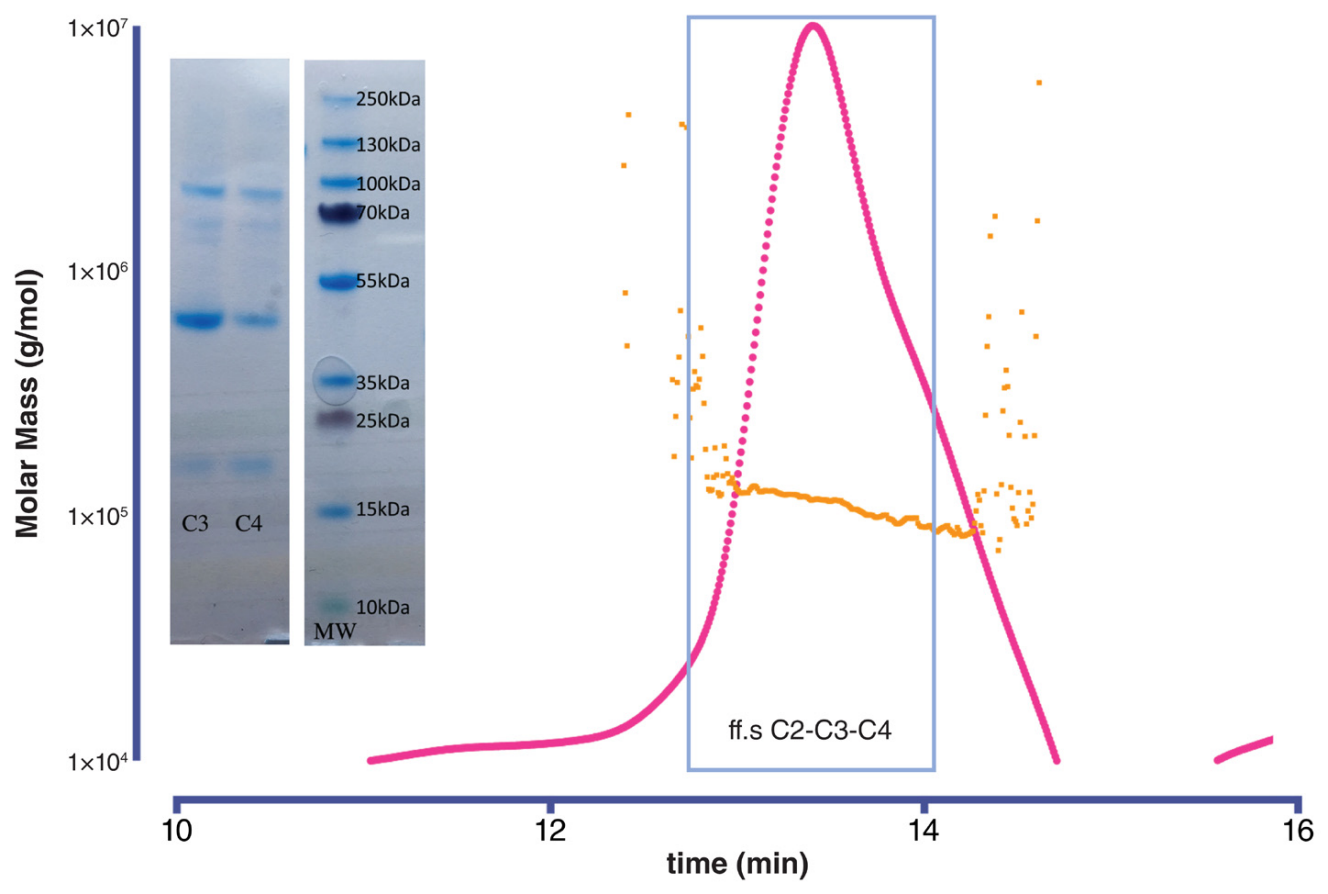
Size exclusion chromatography multiangle light scattering (SEC-MALS) elution profile of *Hs*GAPDH. Elution profile of the *Hs*GAPDH sample run on the SEC S200 10/300 column interfaced with the 18-angle MALS light scattering detector (Dawn HELEOS II ^®^) coupled with a differential refractive index detector (Optilab ^®^ T-rEX) (Wyatt Technology). The fractions C3-C4 eluting between 13 and 14 mL (boxed) correspond to a *Hs*GAPDH tetramer of apparent mass 144 KDa. Inset: the SDS-PAGE NuPAGE Bis-Tris 4-12% gel (Thermo Fisher NP0322PK2), run at 200 V for 30’ in MES buffer, stained in Simply Blue™ SafeStain (Thermo Fisher LC6060) for 1 h and destained with water. The unedited gel has been deposited together with the Open Laboratory Notebook page (see *Underlying data* ^18^).

A volume of 30 *µ*L of the *Hs*GAPDH obtained from the S200 SEC run was diluted to 500 *µ*L in filter-sterilised and degassed buffer HEPES (Sigma Aldrich H3375) 20 mM pH7.5, NaCl 100 mM, TCEP 1mM and injected onto a Superdex S200 10/300 column equilibrated in the same buffer. The mass of the sample was detected on elution with an 18-angle multiangle light scattering detector (Dawn ^®^ HELEOS ^®^ II) coupled with a differential Refractive Index detector (Optilab ^®^ T-rEX) (Wyatt Technology). Figure 2 illustrates the results of the run. The three fractions C2-C3-C4 eluting between 12.5 and 14 mL were pooled and concentrated to a volume of 45 *µ*L - and a concentration of 3.0 mg/mL.

### Protein crystallisation

Vapour diffusion crystallisation 200 nL sitting drops were set up using a Mosquito crystallisation robot (SPT Labtech), mixing *Hs*GAPDH in mother liquor:protein ratioes 1:1 (drop 1) and 1:2 (drop 2), with the 96-conditions MORPHEUS crystallisation screen (Molecular Dimensions, ^26, 27^), and the drops were left equilibrating at 18°C. **P2**_1_ **Forms A and B:** the protein from the S200 SEC run was set up for crystallisation at a concentration of 18.8 mg/mL. Form A: these crystals grew in drop 2 equilibrated against condition H9 of the MORPHEUS crystallisation screen (Molecular dimensions, ^26, 27^): 0.1 M Amino acids solution (DL-Glutamic acid, DL-Alanine, Glycine, DL-Lysine, DL-Serine); 0.1 M Buffer System 3 (Tris (base), bicine pH 8.5); 30% v/v Precipitant Mix 1 (40% v/v PEG 500MME, 20% w/v PEG 20000). Form B: these crystals grew in drop 2 equilibrated against condition F9 of the MORPHEUS crystallisation screen ^26, 27^: 0.12M monosaccharides solution (D-Glucose, D-Mannose, D-Galactose, L-Fucose, D-Xylose, N-Acetyl-D-Glucosamine); 0.1M Buffer System 3 (Tris (base), bicine pH 8.5) 30% v/v Precipitant Mix 1 (40% v/v PEG 500MME, 20% w/v PEG 20000). **P2**_1_ **Forms C and D:** the protein from the size exclusion chromatography multiangle light scattering (SEC-MALS) run was set up for crystallisation at a concentration of 3.0 mg/mL. Both form C and from D grew in drop 1 equilibrated against the F1 condition of the MORPHEUS crystallisation screen ^26, 27^: 0.12 M monosaccharides solution (D-Glucose, D-Mannose, D-Galactose, L-Fucose, D-Xylose, N-Acetyl-D-Glucosamine); 0.1 M Buffer System 3 (Tris (base), bicine pH 6.5) 30% v/v Precipitant Mix 1 (40% v/v PEG 500MME, 20% w/v PEG 20000).

### X-ray data diffraction collection and processing

All crystals were cryo-cooled by plunging them into liquid nitrogen. X-ray diffraction data were collected at beamline I03 of the Diamond Light Source in Harwell, England, UK, with an X-ray beam of wavelength *λ*=0.97622 Å and size 80x20 *μ*M. Other data collection parameters are listed in Table 2. X-ray diffraction data were processed with the autoPROC suite of programs version 1.0.5 ^28^ with the following command line options: process -h5 Datapath/Data_master.h5 -M HighResCutOnCChalf -d DataOutputDirectory. An alternative Open Source suite that would enable X-ray diffraction data processing in equivalent ways is xia2 ^29^ running DIALS ^30^.

**Table 2.** Human GAPDH crystal X-ray diffraction data collection parameters and data processing statistics. Values in parentheses refer to the highest resolution shell.

	Form A	Form B	Form C	Form D
PDB ID	6YND	6YNE	6YNF	6YNH
Det. dist., d *_max_* (mm, Å)	198.35, 1.5	288.19, 2.0	253.03, 1.8	356.69, 2.4
Photon flux (photons/s)	8.84×10 ^11^	8.88×10 ^11^	8.85×10 ^11^	3.88×10 ^12^
Transmission	25%	25%	25%	100%
Number of images	3,600	3,600	3,600	3,600
Oscillation range ( ^◦^)	0.10	0.10	0.1	0.25
Exposure time (s)	0.05	0.05	0.03	0.013
Space Group	P2 _1_	P2 _1_	P2 _1_	P2 _1_
Cell edges: a,b,c (Å)	81.88, 124.45, 141.99	81.79, 124.65, 79.64	87.14, 111.43, 135.94	87.02, 111.30, 69.74
Cell angle *β* ( ^◦^)	99.38	117.04	96.02	98.33
Resolution Range (Å)	93.04-1.52 (1.71-1.52)	72.85-1.85 (2.05-1.85)	135.18-2.39 (2.74-2.39)	86.10-2.62 (2.89-2.62)
R *_merge_*	0.08 (1.07)	0.226 (1.488)	0.27 (1.28)	0.40 (2.47)
R *_meas_*	0.09 (1.16)	0.245 (1.618)	0.29 (1.38)	0.41 (2.54)
Observations	2,029,459 (100,354)	568,580 (26,433)	418,478 (20,849)	496,488 (24,336)
Unique observations	295,270 (14,763)	81,465 (4,074)	59,243 (2,962)	28,207 (1,409)
Average I/ *σ*(I)	11.7 (1.7)	6.5 (1.5)	6.5 (1.6)	9.0 (1.5)
Completeness	69.4 (12.2)	67.6 (12.8)	58.2 (8.7)	71.3 (14.0)
Multiplicity	6.9 (6.8)	7.0 (6.5)	7.1 (7.0)	17.6 (17.3)
CC _1/2_	0.997 (0.608)	0.991 (0.456)	0.986 (0.591)	0.990 (0.464)

### Structure determination and refinement

**P2**_1_ **Form A:** initial structure factor phases were computed by molecular replacement, searching for eight copies of the *Hs*GAPDH monomer in PDB ID 1U8F in space group P2 _1_ using the program CCP4-Molrep version 11.7.02 ^31^ with all default parameters. The eight copies of the *Hs*GAPDH monomer are arranged in two tetramers in the asymmetric unit. Initial automated water addition and positional and individual B-factor refinement were carried out in autoBUSTER, version 2.10.3 ^32, 33^. An alternative Open Source piece of software that would enable refinement against X-ray diffraction data in equivalent ways is Vagabond ^34^. autoBUSTER was run with the following command line options: refine -m Data.staraniso_alldata-unique.mtz -p Model.pdb -autoncs -Seq GAPDH.seq -d OutputDir -l XPE.grade_PDB_ligand.cif ExcludeBadContacts=’EXCLUDE *|203:* *|203:*’ AutomaticFormfactorCorrection=yes -l CSU.grade_PDB_ligand.cif. Automated non-crystallographic restraints were used throughout ^35^, including water molecules (assigned to each chain using CCP4-Sortwater version 7.0.078, with all default parameters). At each catalytic Cys152 site, a 0.5:0.5 occupancy ratio mixture of Cys and Cys S-sulfonic acid was initially modelled in unbiased Fo-Fc residual density (see Figure 5). At each Cys152 site, occupancies for Cys and Cys S-sulfonic acid were then refined under the constraint that they sum up to 1.000±0.005. Crystal form A was deposited in the Protein Data Bank (PDB) with ID code 6YND. Refinement statistics are reported in Table 3. **P2**_1_ **Forms B,C and D:** initial structure factor phases were computed by molecular replacement, searching with the P2 _1_ Form A tetramer (PDB ID 6YND) in space group P2 _1_, placing one tetramer per asymmetric unit in P2 _1_ Forms B and D and two tetramers per asymmetric unit in P2 _1_ Form C, using the program CCP4-Molrep ^31^ version 11.7.02, with all default parameters. The same refinement protocol and restraints were used as described for P2 _1_ Form A, but with additional external secondary structure restraints ^35^ to the highest resolution P2 _1_ Form A structure, using the extra command line flag to -ref 6YND.pdb when running autoBUSTER, version 2.10.3 ^32, 33^. An alternative Open Source piece of software that would enable refinement against X-ray diffraction data in equivalent ways is Vagabond ^34^. Crystal forms B, C and D were deposited in the PDB with ID codes 6YNE, 6YNF and 6YNH, respectively. Refinement statistics are reported in Table 3.

**Table 3.** Human GAPDH crystal structures refinement statistics. Values in parentheses refer to the highest resolution shell, unless otherwise specified.

	Form A	Form B	Form C	Form D
PDB ID	6YND	6YNE	6YNF	6YNH
Space group (Z)	P2 _1_ (16)	P2 _1_ (8)	P2 _1_ (16)	P21 (8)
Resolution range	140.10-1.52(1.63-1.52)	72.85-1.85 (1.98-1.85)	135.18-2.39 (2.62-2.39)	86.10-2.62 (2.72-2.62)
Reflex.s working set	280,585 (5,242)	77,398 (1,536)	56,303 (1,124)	25,751 (545)
Reflex.s free set	14,686 (332)	4,059 (94)	2,940 (61)	1,459 (20)
R,R _*f ree*_	0.182,0.199 (0.209,0.225)	0.190,0.207 (0.218,0.220)	0.179,0.219 (0.222,0.347)	0.176,0.215 (0.231,0.359)
Rmsd _*bonds*_ (Å)	0.008	0.008	0.009	0.008
Rmsd _*angles*_ (◦)	1.02	1.05	1.04	1.03
Ramachandran fav.	97.4% (2,697/2,768)	97.5% (1,304/1,338)	97.0% (2,586/2,667)	97.5% (1,300/1,333)
Ramachandran allow.	99.7% (2,760/2,768)	99.7% (1,334/1,338)	99.5% (2,653/2,667)	99.7% (1,329/1,333)
Tetramers	(A,C,E,H) and (B,D,F,G)	(B,D,F,G)	(A,C,E,H) and (B,D,F,G)	(B,D,F,G)
Occ. Cys-SO _3_H	A:0.13 B:0.15	N/A	A:0.5 B:0.41	B:0.41
	C:0.09 D:0.18	N/A	C:0.46 D:0.33	D:0.42
	E:0.13 F:0.15	N/A	E:0.38 F:0.43	F:0.40
	G:0.15 H:0.08	N/A	G:0.42 H:0.72	G:0.35
Prot.(Wat.) Atoms	20,428 (1,639)	10,108 (569)	20,152 (726)	10,164 (132)
〈B〉 _*prot*_ (〈B〉 _*wat*_) (Å ^2^)	27.26 (33.12)	29.40 (36.17)	39.74 (22.93)	45.38 (29.13)

### Mass spectrometry

Protein was purified by 1D-PAGE before Coomassie staining and excision for proteomic analysis. Protein was digested in-gel with the addition of 20 ng sequencing grade trypsin (Promega V5111). No reduction or alkylation was performed during digestion to help preserve the native state of oxidation. Resulting peptides were analysed over a 1 h liquid chromatography–mass spectrometry acquisition with elution from a 50 cm EasyNano C18 column as detailed in 36 onto a Thermo Orbitrap Fusion Tribrid mass spectrometer. Spectra were acquired in data dependent acquisition mode with sequential fragmentation of all selected peptide precursors using both higher-energy collisional dissociation and electrontransfer dissociation (ETD). All spectra were acquired in the Orbitrap mass analyser with internal calibration from the ETD reagent.

**Figure 3.**
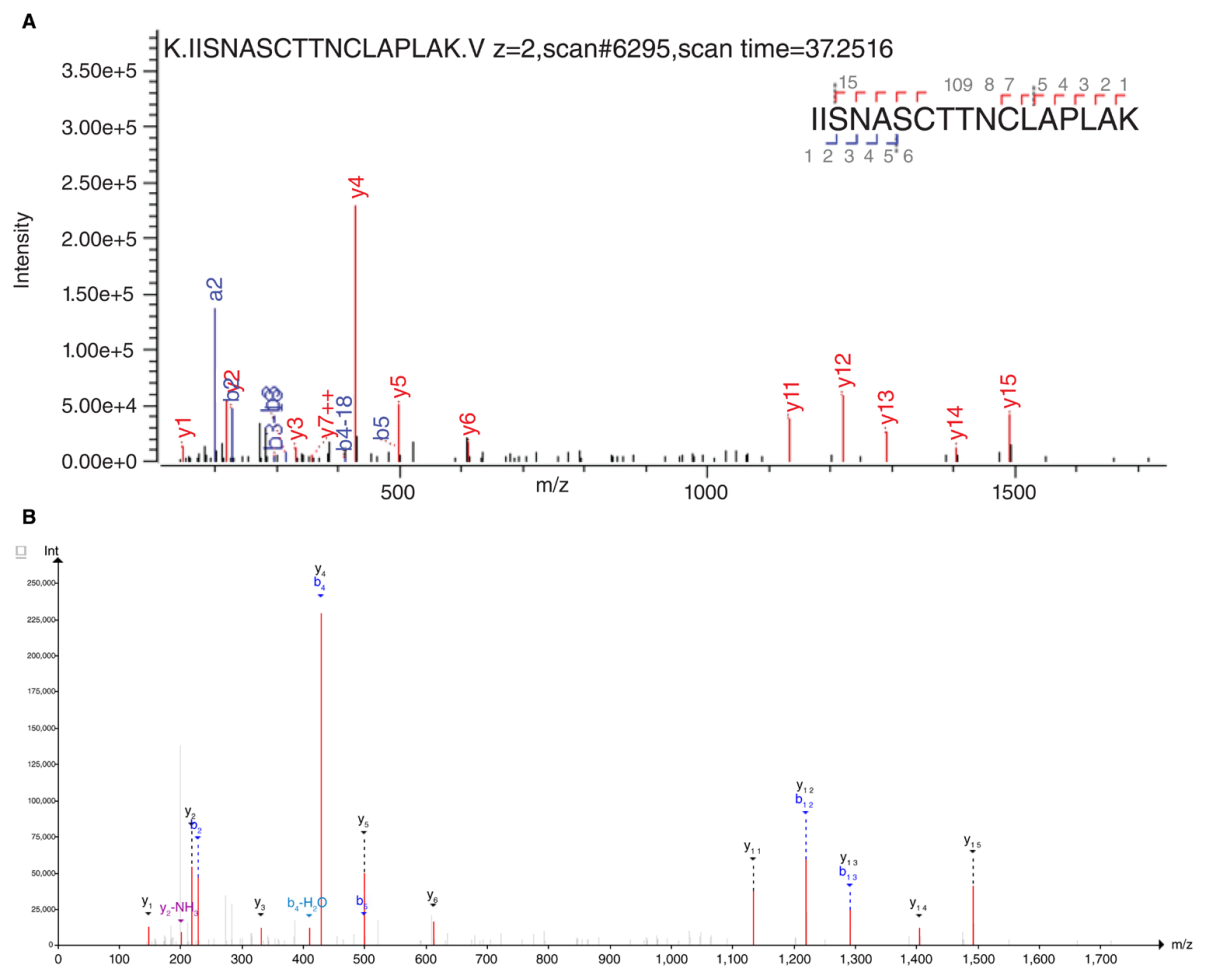
Product ion spectrum of GAPDH-derived tryptic peptide ^146^IISNASCTTNCLAPLAK ^162^ lacking Cys modification. Product ions are annotated with theoretical b- and y- ions (fragment ions that appear to extend from the amino- or carboxy-terminus of a peptide, respectively) from the peptide assignment IISNASCTTNCLAPLAK. **A**: data analysed using Mascot ^37^, PEAKS ^38^ and Byonic™ ^39^ search engines, with all default parameters. **B**: data analysed using MSConvert ^40^, SearchGui ^41^ and PeptideShaker ^42^.

**Figure 4.**
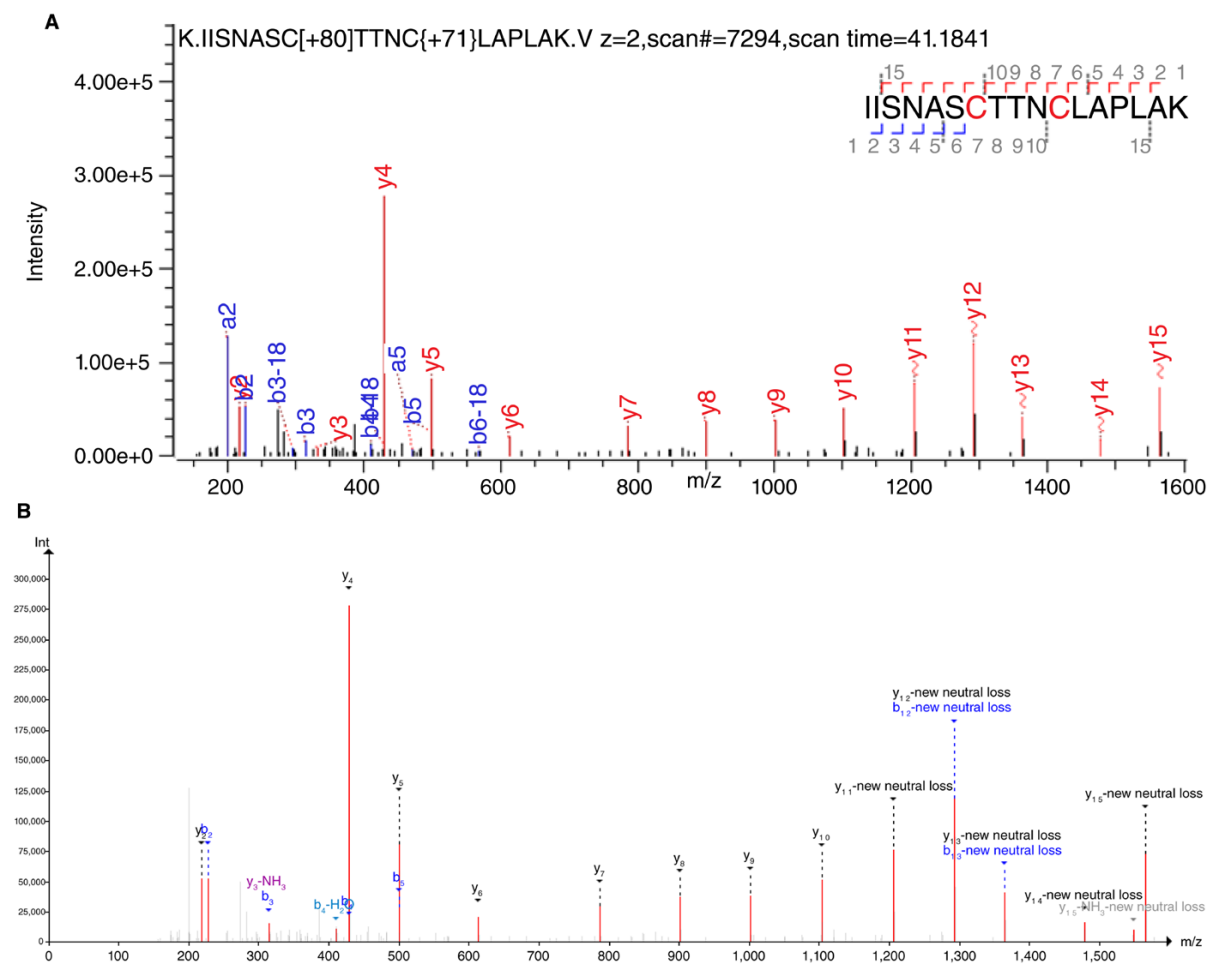
Product ion spectrum of GAPDH-derived tryptic peptide ^146^IISNASCTTNCLAPLAK ^162^. A precursor equating to a mass of 1869.8623 Da was selected and fragmented by HCD. Resulting ions are annotated with theoretical b- and y- ions from the peptide assignment IISNASC(+79.9568 Da)TTNC(+71.036 Da)LAPLAK using the Byonic™ search engine. Annotation of acrylamide adduct modification (+71.036 Da) at Cys156 is implicitly localised from the m/z difference between the product ions y6 and y7. Cys-S-sulfonic acid modification at Cys152 is implied from the difference in mass between the precursor selected and the resulting y11-15 m/z values observed as neutral mass products labelled with "~". The measured neutral mass difference is 79.9568 Da, equating to a mass error of 0.018 mDa for S-sulfonic acid modification. **A**: data analysed using Mascot ^37^, PEAKS ^38^ and Byonic™ ^39^ search engines, with all default parameters. **B**: data analysed using MSConvert ^40^, SearchGui ^41^ and PeptideShaker ^42^.

Product ion spectra were searched against the expected sequence of *Hs*GAPDH (Uniprot P04406) appended to a custom in-house database. All pieces of software were run with default parameters, with the exception that propionamide (+71.037114) and dehydro (-1.007825 Da) were added as manually defined variable modifications. The analysis was carried out using Mascot version 2.6.1 (Matrix Science) ^37^, PEAKS Studio X+,10.5 (Bioinformatics Solutions Inc.) ^38^ and Byonic™ 3.0 (Protein Metrics Inc) ^39^ search engines ( Figure 3A and Figure 4A). The same data analysis was also carried out with freeware, using a combination of MSConvert version 3.0 ^40^, SearchGui version 3.3.18 ^41^ and PeptideShaker version 1.16.45 ^42^ ( Figure 3B and Figure 4B). The resulting peptide assignments are equivalent to the ones obtained with the commercial pieces of software, see Figure 3 and Figure 4. Searching specified a precursor tolerance of 3 ppm and a fragment ion tolerance of 0.02 Da. Initial searches included variable modification of: S-Oxy Cysteine ( Figure 1A), Cysteine S-sulphinic acid ( Figure 1B), and Cysteine S-sulphonic acid ( Figure 1D). Searches were then expanded in PEAKS ^38^ to include 313 of the most frequently observed proteomic modifications and in Byonic™ ^39^ to include disulfide bonding and wildcard mass addition to Cys.

Peptide spectra matches were filtered to p-values (or equivalents) of <0.05. Individual spectra were manually inspected for site localisation or ambiguity before reporting. Global protein coverage of over 90% was achieved. Individual post-translational modification (PTM) modified spectra were manually inspected for site localisation specificity and potential PTM ambiguity before reporting.

## Results

Since the first crystal structures of the lobster GAPDH enzyme ^43– 45^ several crystal structures of prokaryotic and eukaryotic GAPDHs have been determined. At the time of this writing, the PDB contains more than 142 GAPDH entries, 11 of which are of human GAPDH isoforms (see Table 1). All human GAPDH crystal structures described so far contain tetramers, sitting in crystal sites of three different symmetries: point group 1 (four copies of the monomer all slightly different from each other); point group 2 (dimer of dimers); and point group 222 (four identical copies in a tetramer of exact 222 symmetry).

*Hs*GAPDH crystals of form A and form B grew from the *Hs*GAPDH protein sample purified from the supernatant of HEK293F cells by two chromatography steps (IMAC+SEC); crystals of forms C and form D grew from the same *Hs*GAPDH protein sample after an extra SEC-MALS chromatography step, see Figure 2. Our *Hs*GAPDH crystal structures all contain tetramers of point group 1 (two tetramers per asymmetric unit in forms A and C, and one tetramer per asymmetric units in forms B and D). Overall, the four novel crystal forms contain 24 crystallographically independent observations of the *Hs*GAPDH molecule, adding to the 29 ones already present in the PDB at the time of this writing (see Table 1). The overall rmsd _*Cα*_ is 0.170 Å across these 24 crystallographically independent copies of *Hs*GAPDH.

The protein sample from which crystal forms C and D were grown was analysed by mass spectrometry. In addition to peptides corresponding to unmodified Cys152 (see Figure 3), we detected ions derived from fragmentation of the Cys152 containing tryptic peptide ^146^IISNAS **C**TTN **C**LAPLAK ^162^, which suggests Cys152 S-sulfonic acid modification ( Figure 4). Annotation is complicated by the presence of a further modification at Cys156 with a mass addition of +71.036 Da, as directly observed in the fragmentation spectrum by the mass spacing between the y-6 and y-7 ions ( Figure 4). The +71.036 Da mass addition at Cys156 is best annotated as acrylamide adduct (propionamide) imparted during PAGE separation ^48^ rather than being a native modification at this residue. The measured mass of the peptide was 1869.8623 Da and the theoretical mass for the unmodified peptide is 1718.8695 Da. Including the annotated acrylamide adduct, this leaves 79.9568 Da unaccounted for, which is an extremely close match to S-sulfonic acid modification of Cys (+79.9568 Da). A potential ambiguity for the assignment is the similarity in mass of phosphorylation (+79.966331); however, phosphorylation would equate to mass error of 9.5 mDa as opposed to just 0.018 mDa for S-sulfonic acid. Unlike in the case of the acrylamide adduct, the +79.9568 Da mass addition cannot be observed in any of the individual b- or y-ions in the fragmentation spectrum ( Figure 4), implying complete neutral loss of the moiety between precursor selection and fragmentation. Although neutral loss is frequently observed with Ser and Thr phosphorylation, it is rarely seen with 100% efficiency, further suggesting sulfation of Cys152 as the most credible assignment. Relative quantification of modified *versus* unmodified peptide forms is not possible based on the mass spectrometry data alone, due to unknown but likely significant differences in relative response resulting from the changes in the physico-chemical properties of the Cys152-containing ions upon Cys-S-sulfation.

**Figure 5.**
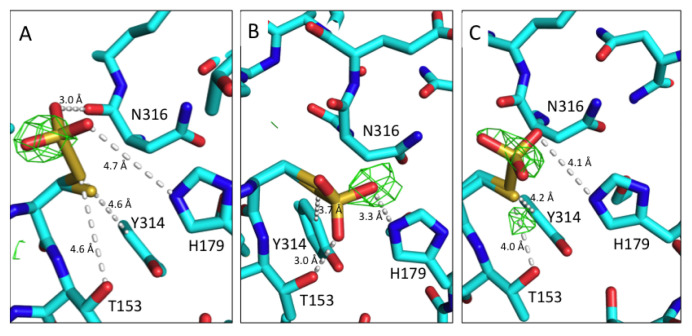
Fo-Fc difference crystal electron density map around *Hs*GAPDH Cys152 residues. Representative *Hs*GAPDH monomers in crystal form C (PDB ID 6YNF) are shown: **A**: monomer B; **B**: monomer E; **C**: monomer G. The 2.5 *σ* contour of the unbiased Fo-Fc map is depicted as a green mesh (the map was calculated with phases from the model before the Cys-S-sulfonic acid modification was built). Residue *Hs*GAPDH 152 was modelled and refined as a 0.28:0.72 superposition of Cys:Cys-S-sulfonic acid. C atoms: cyan; N atoms: blue; O atoms: red; S atoms: yellow. Selected non-bonding interactions between the modified Cys and neighbouring residues are represented as white dashed lines with distances in Å. The Figure was made in PyMOL ^46^. An alternative Open Source piece of software is USF Chimera ^47^.

**Figure 6.**
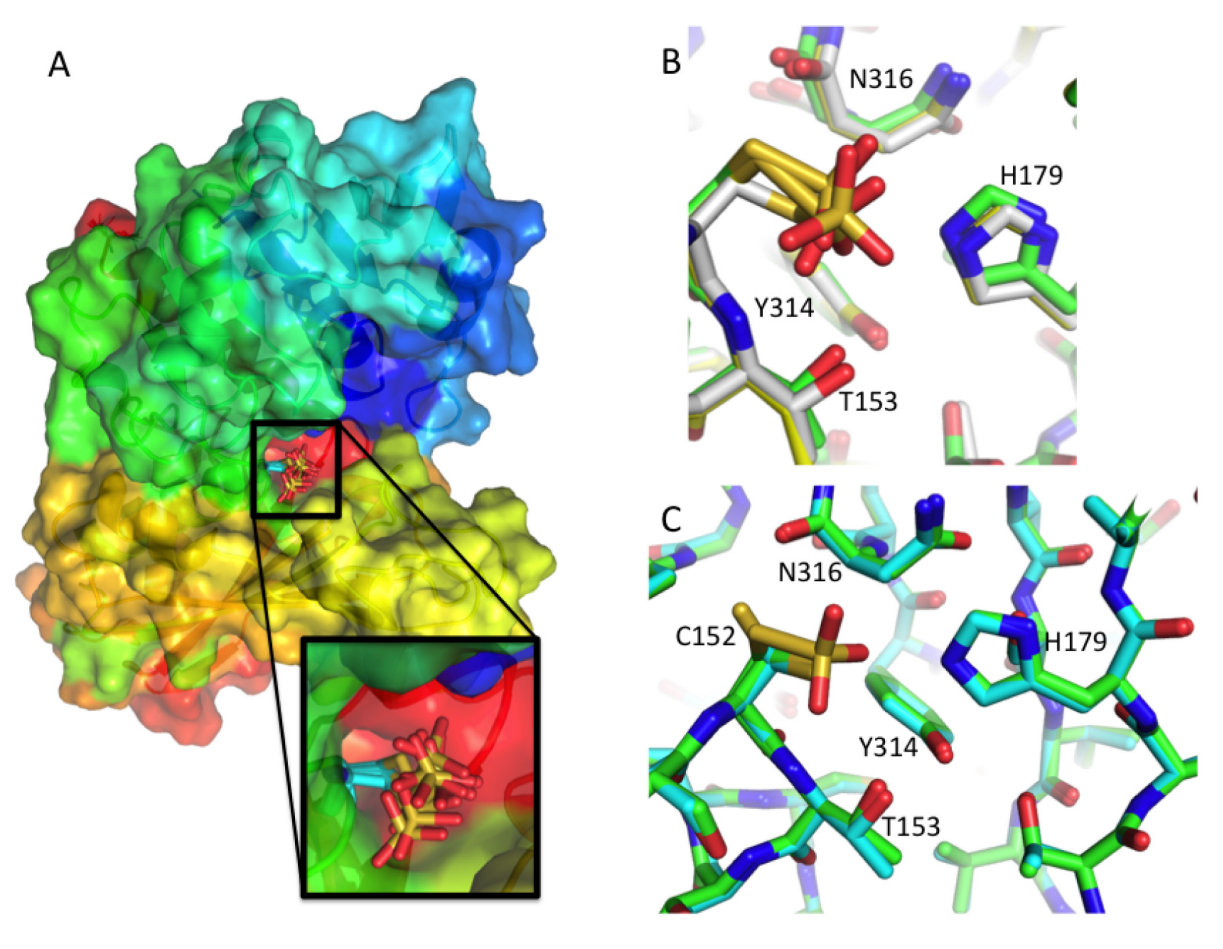
Cys152-S-sulfonic acid modification of *Hs*GAPDH . N atoms: blue; O atoms: red; S atoms: yellow. **A**: the Cys152-S-sulfonic acid modification in the 8 copies of *Hs*GAPDH in crystal form C. The GAPDH monomer is in surface representation and coloured blue to red from N- to C-terminus. The inset shows the Cys152 S-sulfonic acid chains adopting slightly different conformations in different molecules in the asymmetric unit. **B**: superposition of the active site of Cys152-S-sulfonic acid modified *Hs*GAPDH (chain H in crystal form C, green C atoms) with two copies of Cys152-S-sulfonic acid modified *Streptococcus pneumoniae* GAPDH ( *Sp*GAPDH, PDB ID 5M6D, chain "A", white C atoms; chain "B", yellow C atoms). **C**: superposition of the active site of Cys152-S-sulfonic acid modified *Hs*GAPDH (chain H in crystal form C, green C atoms) with native *Hs*GAPDH (chain "O" of PDB ID 1U8F, cyan C atoms). The side chain of the catalytic Cys152 of native *Hs*GAPDH in PDB ID 1U8F was modelled and refined as a superposition two alternate conformations. The Figure was made in PyMOL ^46^. An alternative Open Source piece of software is USF Chimera ^47^.

Peaks suggesting Cys152 S-sulfonic acid modification are also visible in the initial crystal electron density Fo-Fc difference maps of crystal forms A, C and D ^1^, contoured at 2.5 *σ* level or higher, in close proximity to the Cys152 *γ* sulfur atom of most *Hs*GAPDH chains in the asymmetric unit (see Figure 5). The orientation of the Cys-S-sulfonic acid moiety in the active site is not unique, but it varies from chain to chain across the three crystal forms. Figure 6A illustrates the modification in the eight copies of *Hs*GAPDH in crystal form C. The Cys152 S-sulfonic acid chain makes only loose non-bonding contacts to residues in the active site (see also Figure 5). Modelling of the Cys152 residue as a superposition of Cys and Cys-S-sulfonic acid with variable occupancies in crystal forms A, C and D enables estimation of the extent to which the crystals contain the Cys152 modification. The average occupancy for the Cys152-S-sulfonic acid modification over these 20 crystallographically independent copies of *Hs*GAPDH is 0.31±0.17 (see Table 3). The *Hs*GAPDH monomer with the largest occupancy for the modification (Occ _*C ys*− *S*− *SO*3_=0.72) is monomer H in crystal form C. This predominantly Cys-S-sulfonated *Hs*GAPDH monomer superposes with an rmsd _*C α*_=0.423 Å (over 334 C _*α*_ atoms) with the *Streptococcus pneumoniae* GAPDH monomer carrying the same modification ^17^, see Figure 6B. When comparing the modified structure to the one of *Hs*GAPDH with unmodified Cys152 (PDB ID 1U8F) no significant local changes to active site side chains are observed either (rmsd _*Cα*_=0.127 over 333 C _*α*_ atoms), see Figure 6C. None of the *Hs*GAPDH molecules in the crystals contain NAD ^+^ nor any bound ligand in the active site.

## Discussion and conclusions

GAPDH is mainly a cytoplasmic enzyme, but it has been reported to moonlight as a cell surface and/or extracellular enzyme in healthy human cells ^49, 50^, in human cells subjected to stresses such as micronutrient starvation, hypoxia, infection, and cancer ^50– 54^, and to act as a extracellular effector in fungi and bacteria ^55– 62^. For recent reviews on GAPDH inhibitors and their multiple roles in several pharmacological applications see 63, 64.

In our study, *Hs*GAPDH was purified from the supernatant of HEK293F cells treated with the mannosidase inhibitors kifunensine and kept for 4 days at 37°C after transfection with a DNA plasmid for recombinant expression of an endoplasmic reticulum glycoprotein. Although kifunensine is toxic to cells only in concentrations higher than the one used (5 *μ*M), during the 4 days between transfection and harvesting of the supernatant the experimental conditions are likely harsher than the basal levels of oxidative stress in the HEK293F cell culture media ^65^.

Stable oxidation of the GAPDH catalytic cysteine has been observed in a number of studies, for example to Cys sulfenic acid mediated by thiolate oxidation ^66^: the modification is reversible but it confers the modified cysteine electrophilic properties similar to the ones of sulfenyl halides ^67^. For example, GAPDH catalytic cysteine modification to sulfenic acid converts the enzyme from dehydrogenase to an acyl phosphatase ^68– 70^. In the presence of excess oxidant, further oxidation of sulfenic acid to sulfinic acid or sulfonic acid is possible. However, in contrast to GAPDH catalytic Cys oxidation to sulfenic acid, which is reversible, oxidation to sulfonic acid leads to irreversible enzyme inactivation and Cys S-sulfonation is generally considered an irreversible form associated with protein misfolding, degradation, and pathology ^67^. In particular, S-sulfonated GAPDH has been reported to translocate to subcellular domains where it does not normally occur, where it may stimulate a “gain of function” that could provoke apoptosis ^49^.

It is unclear whether the Cys S-sulfonic acid modification we observe in *Hs*GAPDH purified from the supernatant of HEK293F cells represents a physiological response of the cells to the recombinant secreted protein expression system conditions, or whether it is simply the outcome of a protracted oxidative environment on the pool of extracellular enzyme instead. GAPDH allostery is mediated by intra-dimer contacts (the active site of one dimer subunit is in close proximity to residues in the neighbouring subunit). The observation that *Hs*GAPDH tetramers pack in the crystals in ordered fashion even in presence of partial Cys-S-sulfonic acid modification, together with the overall structural similarity of the Cys S-sulfonated and native monomeric enzymes, suggests that the secreted *Hs*GAPDH is constantly inactivated by catalytic Cys S-sulfonation in the expression system’s oxidising extracellular environment. Of course, it is also possible that the relative amounts of oxidised vs. reduced GAPDH could vary during protein purification (after harvesting the cells’ supernatant) due to changes in the ionic strength and pH; all buffers used were degassed (minimising O _2_ content) but contain 1 mM Tris (2-Carboxyethyl) phosphine (TCEP), a reducing agent that prevents intermolecular disulphide_bond-mediated oligomerisation of GAPDH. The crystallisation conditions do not nominally contain redox active ingredients but crystal forms A,B grow at pH 8.5, while forms C,D grow at pH 6.5. MS characterisation of GAPDH samples purified at sequential stages during the expression/ purification procedure would elucidate the time-dependence of the enzyme catalytic Cys residue redox composition in the chosen experimental conditions.

## Data availability

### Underlying data

X-ray data of forms A, B, C and D at Protein Data Bank, Accession numbers 6YND, 6YNE, 6YNF and 6YNH:

https://identifiers.org/pdb:6YND

https://identifiers.org/pdb:6YNE

https://identifiers.org/pdb:6YNF

https://identifiers.org/pdb:6YNH

Mass spectrometry datasets at MassIVE, Accession number MSV000085325: https://identifiers.org/ massive:MSV000085325

Zenodo: Crystal structure determination of Cys-S-sulfonated *Hs*GAPDH from protein purified from the supernatant of HEK293F cells. https://doi.org/10.5281/zenodo.3817277 ^18^

This project contains the following underlying data:

- Gel-Figure3.jpeg (original unedited gel image for Figure 2)

### Extended data

Zenodo: Crystal structure determination of Cys-S-sulfonated *Hs*GAPDH from protein purified from the supernatant of HEK293F cells. https://doi.org/10.5281/zenodo.3817277 ^18^

This project contains the following extended data:

- Copy of the Open Laboratory Notebook (PDF)
- Original unedited gel images for Open Laboratory Notebook (JPG, JPEG and PNG files)

Data are available under the terms of the Creative Commons Attribution 4.0 International license (CC-BY 4.0).
