# Supplementary material for: Partial catalytic Cys oxidation of human GAPDH to Cys-sulfonic acid
Source: Wellcome Open Res. 2020 Aug 25;5:114. Originally published 2020 Jun 1. [Version 2] doi: 10.12688/wellcomeopenres.15893.2 (PMC7422855; doi:10.12688/wellcomeopenres.15893.2)
Supplement: Peer review history

# Peer review: Reviewer response for version 1

**DOI:** 10.21956/wellcomeopenres.17433.r39295

**Published:** 2020-08-11

**Authors:** Chaaya I. Raje

**Affiliations:** 1 National Institute of Pharmaceutical Education and Research, Mohali, Punjab, India

The authors Lia A et al., provide X-ray crystallography data to define the Cys-152-S-sulfonic acid modification observed in human GAPDH. Post translational modifications are known to modulate the localization and function of GAPDH. This study provides critical structural information that can be utilized by other research groups investigating the diverse cellular functions of this multifaceted protein. Overall the study is focused with excellent discussion, it be a significant contribution in this field. No additional experiments or changes are suggested.

Is the work clearly and accurately presented and does it cite the current literature?

Yes

If applicable, is the statistical analysis and its interpretation appropriate?

I cannot comment. A qualified statistician is required.

Are all the source data underlying the results available to ensure full reproducibility?

Yes

Is the study design appropriate and is the work technically sound?

Yes

Are the conclusions drawn adequately supported by the results?

Yes

Are sufficient details of methods and analysis provided to allow replication by others?

Yes

Reviewer Expertise:

Protein multifunctionality, host-pathogen interaction, iron metabolism in M.tuberculosis

I confirm that I have read this submission and believe that I have an appropriate level of expertise to confirm that it is of an acceptable scientific standard.

# Peer review: Reviewer response for version 1

**DOI:** 10.21956/wellcomeopenres.17433.r39378

**Published:** 2020-07-13

**Authors:** Vladimir Muronetz

**Affiliations:** 1 Belozerskiy Research Institute of Physicochemical Biology, Lomonosov Moscow State University, Moscow, Russian Federation

The article "Partial catalytic Cys oxidation of human GAPDH” by A. Lia et al. contains new and important information on the structure of the active site of GAPDH with the catalytic Cys152 oxidized to sulfonic acid. It is no less interesting that it was possible to isolate this form of oxidized GAPDH from the conditioned medium of human epithelial kidney cells subjected to stress. The disadvantage of this article is the lack of information on the enzymatic activity of the isolated enzyme, in which only part of the active site cysteines is oxidized. These data could show the degree of irreversible oxidation of the enzyme and confirm the results of crystallographic studies. It should also be noted that the reducing agent was added only on the stage of chromatographic purification of the enzyme. It is possible that the irreversible oxidation of GAPDH could occur not only during cell cultivation, but also during the preparation of the conditioned medium for purification (collecting, dilution, and filtration) as well as during its application on the column.

 Other comments:

  

1. In the title, it would be better to indicate precisely the product of cysteine oxidation.
2. The phrase "GAPDH catalyses the reversible NAD+-dependent oxidative phosphorylation of n-glyceraldehyde-3-phosphate to 1,3- diphospho-n-glycerate in both glycolysis and gluconeogenesis” is inaccurate, since during gluconeogenesis the reverse reaction takes place.
3. “In multiple mammalian species the sperm isozyme shares about 70% amino acid identity with the somatic isozyme and plays a role in anchoring GAPDHS to the fibrous sheath in the principal piece of the sperm flagellum”. The phrase is incomprehensible. It is unclear, what plays a role in anchoring GAPDHS. You can write that the additional N-terminal fragment of the sperm isoenzyme is necessary for anchoring GAPDHS to the fibrous sheath of the sperm flagellum. 
4. The designations of the cysteine derivatives in Fig. 1 could be supplemented with those accepted in enzymology (S-OH, SO2H, SO3H). The accepted name of the protein is D-glyceraldehyde-3-phosphate dehydrogenase.
5. The Introduction and Discussion are overloaded with references to old articles, but there is not enough references to later works. For example, there are references to the first works of 1969-1972 years on GAPDH isolation and on acylphosphatase activity of GAPDH. 
6.  Methods. Cloning and isolation of recombinant mannosidase is excessively detailed in Methods. It should be emphasized that the aim of this work was to isolate GAPDH from the conditioned medium and indicate what is critical for the isolation of this enzyme (for example, the importance of using reducing agents to prevent the oxidation of GAPDH). The authors did not decipher the name of the used reducing agent (TCEP - Tris (2-Carboxyethyl) phosphine), which complicates the understanding of the article.
7. Discussion and conclusion. “However, in contrast to sulfenic acid, higher oxidation states of catalytic Cys residues lead to enzyme inactivation ” – an incorrect phrase, since the oxidation of the catalytic cysteine to sulfenic acid also leads to disappearance of the dehydrogenase activity, although reversible

Is the work clearly and accurately presented and does it cite the current literature?

Partly

If applicable, is the statistical analysis and its interpretation appropriate?

Yes

Are all the source data underlying the results available to ensure full reproducibility?

Yes

Is the study design appropriate and is the work technically sound?

Yes

Are the conclusions drawn adequately supported by the results?

Yes

Are sufficient details of methods and analysis provided to allow replication by others?

Yes

Reviewer Expertise:

Enzymology

I confirm that I have read this submission and believe that I have an appropriate level of expertise to confirm that it is of an acceptable scientific standard.

# Response

**Published:** 2020-08-13

**Authors:** Pietro Roversi

**Affiliations:** University of Leicester, UK

We thank the referee for the attention devoted to our manuscript, for their informed and thoughtful comments, and for the suggestions. We believe we have addressed all of them and we are confident that the manuscript is improved because of this.

 In the following we respond to this Reviewer comments in a point-by-point format.

*The disadvantage of this article is the lack of information on the enzymatic activity of the isolated enzyme, in which only part of the active site cysteines is oxidized. These data could show the degree of irreversible oxidation of the enzyme and confirm the results of crystallographic studies.*

 We agree with the Reviewer that enzymatic activity measurements ought to correlate with the percentage of residual reduced catalytic Cys in our sample. Of course, as we state in the discussion, “in contrast to sulfenic acid, higher oxidation states of catalytic Cys residues lead to enzyme inactivation and Cys S-sulfonation is generally considered an irreversible form associated with protein misfolding, degradation, and pathology (Reference [65])”, so enzymatic assays of our oxidised GAPDH would likely add to the literature very little that is not known already.

*It should also be noted that the reducing agent was added only on the stage of chromatographic purification of the enzyme. It is possible that the irreversible oxidation of GAPDH could occur not only during cell cultivation, but also during the preparation of the conditioned medium for purification (collecting, dilution, and filtration) as well as during its application on the column.*

 This is a very good point and we have added the following paragraph to the discussion: “ *Of course, it is also possible that the relative amounts of oxidised vs. reduced GAPDH could vary during protein purification (after harvesting the cells’ supernatant) due to changes in the ionic strength and pH; all buffers used were degassed (minimising O _2_ content) but contain 1 mM Tris (2-Carboxyethyl) phosphine (TCEP), a reducing agent that prevents intermolecular disulphide_bond-mediated oligomerisation of GAPDH. The crystallisation conditions do not nominally contain redox active ingredients but crystal forms A,B grow at pH 8.5, while forms C,D grow at pH 6.5. MS characterisation of GAPDH samples purified at sequential stages during the expression/ purification procedure would elucidate the time-dependence of the enzyme catalytic Cys residue redox composition in the chosen experimental conditions.”*

**

*Other comments:*

**

*1. In the title, it would be better to indicate precisely the product of cysteine oxidation.*

 We have modified the title to: “Partial catalytic Cys oxidation of human GAPDH to sulfonic acid.”

*2. The phrase "GAPDH catalyses reversible NAD+-dependent oxidative phosphorylation of n-glyceraldehyde-3-phosphate to 1,3- diphospho-n-glycerate in both glycolysis and gluconeogenesis” is inaccurate, since during gluconeogenesis the reverse reaction takes place.*

 We have modified the phrase as follows: “ *GAPDH is essential for glycolysis and gluconeogenesis; it catalyses the NAD+-dependent oxidative phosphorylation of n-glyceraldehyde-3-phosphate to 1,3- diphospho-n-glycerate (and its reverse reaction).* *The GAPDH-catalysed forward reaction occurs at an important transition point in glycolysis between the enzymatic steps that consume and generate ATP.”*

*3.  “In multiple mammalian species the sperm isozyme shares about 70% amino acid identity with the somatic isozyme and plays a role in anchoring GAPDHS to the fibrous sheath in the principal piece of the sperm flagellum”. The phrase is incomprehensible. It is unclear, what plays a role in anchoring GAPDHS. You can write that the additional N-terminal fragment of the sperm isoenzyme is necessary for anchoring GAPDHS to the fibrous sheath of the sperm flagellum.*

 We have modified this sentence along the Reviewer’s suggestion: “ *In multiple mammalian species the sperm isozyme (GAPDHS) shares about 70% amino acid identity with the somatic isozyme, and possesses an additional 72-residues N-terminal extension. The latter enables association of GAPDHS to the sperm flagellum fibrous sheath, so that the enzyme – together with other glycolytic enzymes - provides a localised source of ATP that is essential for sperm motility.*”

*4. The designations of the cysteine derivatives in Fig. 1 could be supplemented with those accepted in enzymology (S-OH, SO2H, SO3H). The accepted name of the protein is D-glyceraldehyde-3-phosphate dehydrogenase.*

             We have modified the Figure – adding the conventional names for the Cys derivatives. We include the accepted name of the protein in the first paragraph of the paper.

*5. The Introduction and Discussion are overloaded with references to old articles, but there is not enough references to later works. For example, there are references to the first works of 1969-1972 years on GAPDH isolation and on acylphosphatase activity of GAPDH.*

             We include references to early work to give credit to the scientists who first characterised the enzyme. We take the point that it is important to reference more recent work too. We have now included references to two reviews on GAPDH inhibition in order to link to more recent GAPDH work: “ *For recent reviews on GAPDH inhibitors and their multiple roles in several pharmacological applications see ^69,70^.”. Ref. 69: Muronetz VI, Melnikova AK, Barinova KV, Schmalhausen EV. Inhibitors of Glyceraldehyde 3-Phosphate Dehydrogenase and Unexpected Effects of Its Reduced Activity. Biochemistry (Mosc). 2019;84(11):1268-1279. doi:10.1134/S0006297919110051; Ref. 70: Lazarev VF, Guzhova IV, Margulis BA. Glyceraldehyde-3-phosphate Dehydrogenase is a Multifaceted Therapeutic Target. Pharmaceutics. 2020;12(5):416. Published 2020 May 2. doi:10.3390/pharmaceutics12050416*.

*6. Methods. Cloning and isolation of recombinant mannosidase is excessively detailed in Methods. It should be emphasized that the aim of this work was to isolate GAPDH from the conditioned medium and indicate what is critical for the isolation of this enzyme (for example, the importance of using reducing agents to prevent the oxidation of GAPDH). The authors did not decipher the name of the used reducing agent (TCEP - Tris (2-Carboxyethyl) phosphine), which complicates the understanding of the article.*

             The details on the cloning of the recombinant mannosidase are necessary for full reproducibility of the experiment as it was carried out. We have added the full chemical name of TCEP in the place in the text where it first appears and explained the intended reason for its presence in our protein chemistry buffers: *“all buffers (…) contain 1 mM Tris (2-Carboxyethyl) phosphine (TCEP), a reducing agent that prevents intermolecular disulphide_bond-mediated oligomerisation of GAPDH.*

*7. Discussion and conclusion. “However, in contrast to sulfenic acid, higher oxidation states of catalytic Cys residues lead to enzyme inactivation ” – an incorrect phrase, since the oxidation of the catalytic cysteine to sulfenic acid also leads to disappearance of the dehydrogenase activity, although reversible.*

             We have modified the sentence as follows: “ *However, in contrast to GAPDH catalytic Cys oxidation to sulfenic acid, which is reversible, oxidation to sulfonic acid leads to irreversible enzyme inactivation”*
